# Epidemiology of intracerebral hemorrhage: A systematic review and meta-analysis

**DOI:** 10.3389/fneur.2022.915813

**Published:** 2022-09-16

**Authors:** Sai Wang, Xue-Lun Zou, Lian-Xu Wu, Hui-Fang Zhou, Linxiao Xiao, Tianxing Yao, Yupeng Zhang, Junyi Ma, Yi Zeng, Le Zhang

**Affiliations:** ^1^Department of Neurology, Xiangya Hospital, Central South University, Changsha, China; ^2^Department of Spine Surgery, Xiangya Hospital, Central South University, Changsha, China; ^3^Department of Geriatrics, Second Xiangya Hospital, Central South University, Changsha, China; ^4^National Clinical Research Center for Geriatric Disorders, Xiangya Hospital, Central South University, Changsha, China; ^5^Multi-Modal Monitoring Technology for Severe Cerebrovascular Disease of Human Engineering Research Center, Xiangya Hospital, Central South University, Changsha, China

**Keywords:** intracerebral hemorrhage, incidence, risk factors, sex difference, hypertension

## Abstract

**Background:**

Intracerebral hemorrhage (ICH) is associated with high mortality and disability rates. This study aimed to investigate the relationship between sex, age, study year, risk factors, bleeding site, median year of study, and the incidence of ICH.

**Method:**

Literature on the incidence of ICH published on 1 January 1980 and 1 January 2020, was systematically retrieved from PubMed and Embase databases. The random-effects model and subgroup analysis were used to explore the relationship between the incidence of ICH and different ages, sex, bleeding sites, and risk factors.

**Results:**

We summarized the epidemiological changes in ICH in the past 40 years according to 52 studies and found that the total incidence of ICH is 29.9 per 100,000 person-years (95% CI: 26.5–33.3), which has not decreased worldwide. The incidence of ICH in the Asian population is much higher than in other continents. In addition, the incidence of ICH increases with age and differs at the 85-year-old boundary. Men are more likely to develop ICH than women, and the basal ganglia region is the most common area for ICH. Of the 10 risk factors examined in this study, those with hypertension had the highest incidence of ICH, followed by those with excessive alcohol consumption and heart disease.

**Conclusion:**

The prevention and treatment of ICH still need to be improved continuously according to age, sex, risk factors, and other factors, and targeted and normative strategies should be gradually developed in the future.

## Introduction

Intracerebral hemorrhage (ICH) is a subtype of stroke, and the incidence of ICH is second only to ischemic stroke. It changes quickly, fluctuates greatly, has extremely high mortality and disability rates (1–4), and significantly threatens human health and quality of life, especially in developing countries (5).

In recent decades, the incidence and mortality of ICH have been reported in Europe, America, Asia, and other regions. A Norwegian survey of ICH from 1995 to 2012 showed that the ICH's incidence and mortality rate have not changed over the past 17 years (6). A meta-analysis of ICH incidence and mortality conducted 10 years ago showed that ICH's incidence had not decreased between 1980 and 2006 (7). Unfortunately, systematic exploration and trend analysis studies of morbidity and mortality of ICH in Africa are still lacking (7, 8). Regulatory risk factors are the key to preventing and treating ICH, and hypertension is the most common and important modifiable risk factor of ICH (9–11). In addition, smoking (9), drinking (9, 11), and anticoagulant drugs (9) also play important roles in the onset of ICH. However, the incidence of ICH induced by risk factors such as hypertension and alcohol consumption is unclear. The site of ICH is related to different risk factors, and different sites of hemorrhage may have an important impact on the prognosis of ICH; therefore, it is of clinical value to study the incidence of ICH at different sites.

This meta-analysis aimed to determine the trend of ICH's incidence and mortality rate in the past several decades and the incidence of ICH according to sex, age, bleeding site, and associated risk factors. Our study focused on the relationship between the incidence of ICH and different ages, sexes, risk factors, and median age, which may have a profound impact on the prevention and treatment of ICH in the future.

## Methods

### Database search

This study followed the PRISMA 2020 guidelines to conduct this systematic review and meta-analysis. We systematically searched the studies published in the PubMed and Embase databases from 1 January 1990, to 1 January 2020, regarding the incidence of ICH. The keywords: “cerebral hemorrhage”, or “intracerebral hemorrhage”, or “ICH”, or “hemorrhage stroke”, or “stroke”, or “cerebrovascular disease”, and “incidence”, or “epidemiology”. Many epidemiological studies were reviewed, and manual retrieval and classification of references, reviews, meta-analyses, and clinical studies related to the incidence of ICH were conducted. All the included studies were published in English.

### Eligibility criteria

Prospective or population-based studies on the incidence of stroke and ICH were included. Moreover, the definition of ICH is non-traumatic intracerebral parenchymal hemorrhage, confirmed by computed tomography (CT), magnetic resonance imaging (MRI), or autopsy, with a confirmation rate of over 70% (9). Subtypes of stroke, such as ischemic stroke and subarachnoid hemorrhage, were excluded. Studies on stroke and hemorrhagic stroke without describing the incidence of ICH and those that did not directly acquire the incidence of ICH were also excluded. These studies did not provide clear data on the incidence of ICH, which may have interfered with their results. Studies were solely based on the international classification of disease codes, and retrospective studies were excluded, as these are poor indicators of ICH'sincidence (10, 11).

### Data extraction, quality assessment, and subgroup analysis

All the data processed standardized independent extraction and records of studies were characterized as follows: title, author, publication date and publishing journal, research state, research types, population ages, number of years of study, mid-year of study, the incidence of risk factors, bleeding site, and impact factor. These characteristics were used for classification and induction. As there is no recognized gold standard evaluation tool for selecting observational epidemiological surveys, a methodological index for a non-randomized controlled experiment scale (MINORS) was adopted for quality evaluation (12). Two investigators independently conducted the quality assessment and completed the data assessment form. If there were any disputes, they sought guidance and comments from experienced professors to determine whether the data were accepted. All the studies strictly followed the inclusion and exclusion criteria of the meta-analysis.

The incidence of ICH in people under 45 years of age is low; therefore, people younger than 45 years of age are classified into one group. In addition, the number of people older than 85 years also accounted for a small proportion, and they were classified into a separate group. The 45–54 years old age group was used as the reference group in the age analysis, and women were used as the reference group in the sex study. Hypertension, the most common risk factor, was used as the reference group in the risk factor study. The hemorrhage was used as the reference group at the site of bleeding in subtype analysis.

### Statistical analysis

We made full use of the relevant studies to summarize information such as the number of cases and the total number of people under investigation in different studies, and calculated the incidence rate of ICH in each region and converted it into the crude incidence rate of 100,000 person-years. The incidence found by multiple studies was summarized by a two-item randomized effect meta-analysis using the number of ICH cases in each study and the total number of people surveyed as variables. Simultaneously, different age ranges, sexes, bleeding sites, risk factors, and other incidence rates were calculated. Double arcsine conversion was used to calculate the total mixing rate and stabilize the variance. All statistical analyses were performed using STATA 14.0 and SPSS 24.0.

## Results

### Literature search

**Figure 1:**
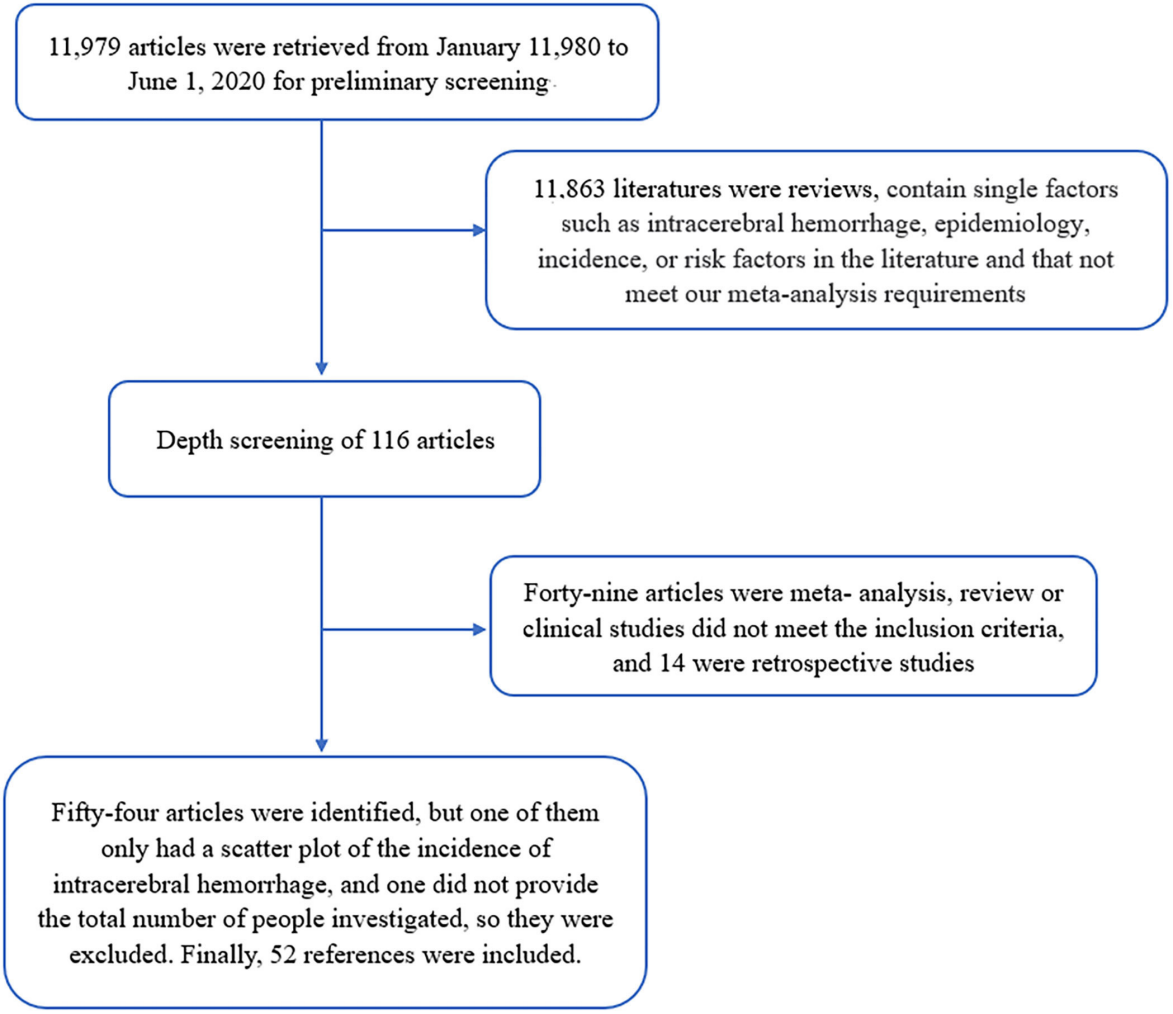
Flow diagram for this systematic review and meta-analysis.

In the preliminary search, 11,979 articles were found; after screening, 11,881 articles that were not strongly related to this study were initially excluded. After many screenings and reviews, we excluded 49 studies that did not meet the inclusion criteria and 14 retrospective studies. In total, 54 relevant studies were identified, of which one only included scatter plots without specific data on ICH incidence, and another did not provide a specific total number of participants, which were excluded to avoid errors. Finally, 52 studies were selected for data analysis and processing (Figure 1).

### Study characteristics and incidence of ICH

**Figure 2:**
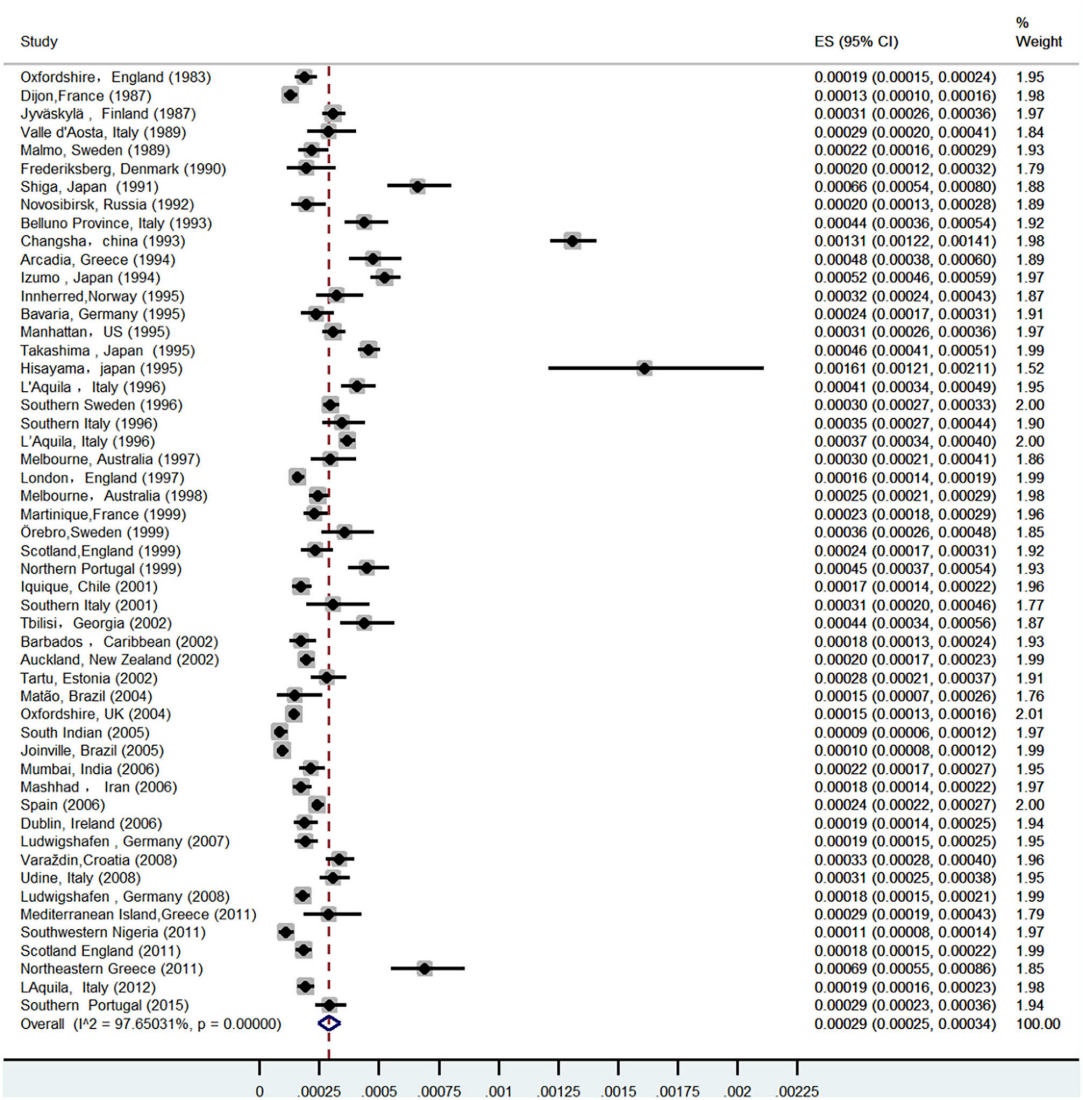
Histogram of the average incidence rate of ICH in all continents.

A total of 52 studies (13–64) from 28 countries were included in this study (Table 1). The total number of people surveyed was 23,933,708, and 6,532 patients had ICH. The incidence of ICH was 29.9 per 100,000 (95% CI: 26.5–33.3) person-years (Figure 2). In total, 28 studies were prospective (13, 15, 17–19, 21, 24, 26, 28, 30, 32–37, 39–41, 43, 46–52, 54, 55, 57), 18 were population-based (14, 20, 22, 23, 25, 29, 42, 44, 45, 47, 53, 55, 59–64), and 4 were community studies (27, 31, 38, 48). In addition, there was also a cohort study (58) conducted over three different periods in Japan, but only data from the third-period cohort study met the criteria of our study.

**Table 1:** Basic information of all included studies.

**Published year of study**	**Ages**	**Study countries and regions**	**Mid-year of study**	**Numbers of patients**	**Person-year**	**Time of study (year)**	**Incidence ratio men vs. women (95% CI)**
1990 (13)	All ages	Oxfordshire, England	1983	66	345,948	4	0.76 (0–1.72)
1991 (14)	All ages	Dijon, France	1987	87	678,560	5	1.57 (1.2–1.94)
1992 (15)	All ages	Valle d'Aosta, Italy	1989	33	114,325	1	…
1992 (16)	All ages	Jyväskylä, Finland	1987	158	510,545	4.3	1.14 (0–2.42)
1992 (17)	All ages	Frederiksberg, Denmark	1990	17	85,611	2	…
1992 (18)	All ages	Malmo, Sweden	1989	51	232,448	…/1	…
1995 (19)	All ages	Belluno, Italy.	1993	93	211,389	1	0.67 (0–1.45)
1995 (20)	All ages	Novosibirsk, Russia	1992	31	158,234	1	…
1997 (21)	All ages	L'Aquila, Italy	1996	122	297,838	5	1.13 (0.78–1.48)
1997 (22)	≥15	Innherred, Norway	1995	45	138,590	2	…
1998 (23)	≥18	Southern Greece	1994	77	161,548	2	2.05 (1.33–2.83)
1998 (24)	All ages	Bavaria, Germany	1995	48	202,900	2	0.86 (0.52–1.2)
1999 (25)	≥35	Shiga, Japan	1991	98	148,370	5	0.94 (0.57–1.31)
2000 (26)	All ages	Southern Sweden	1996	341	1,140,000	1	1.35 (0.78–1.93)
2000 (27)	All ages	Izumo, Japan	1994	267	509,124	6	1.25 (0.54–1.97)
2001 (28)	All ages	Martinique, France	1999	83	360,000	1	…
2001 (29)	All ages	Melbourne, Australia.	1997	40	133,816	1	1.96 (1.1–2.82)
2002 (30)	All ages	London, England	1997	151	938,132	4	…
2002 (31)	All ages	Örebro, Sweden	1999	44	123,503	1	…
2003 (32)	All ages	Southern Italy	1996	62	179,186	1	1.25 (0.5–2.0)
2004 (33)	All ages	Changsha, China	1993	722	551,163	15	1.89 (1.34–2.45)
2004 (34)	All ages	Tbilisi, Georgia	2002	62	140,926	2.7	0.62 (0–1.97)
2004 (35)	All ages	Barbado, Caribbean	2002	42	239,068	1	…
2005 (36)	All ages	Iquique, Chile	2001	69	396,712	2	1.79(0–3.8)
2005 (37)	≥20	Manhattan, US	1995	155	501,618	4	…
2005 (38)	All ages	Scotland, England	1999	50	212,704	2	…
2006 (39)	≥15	Auckland, New Zealand.	2002	177	897,882	1	0.75 (0–1.62)
2007 (40)	All ages	Matão, Brazil	2004	11	75,053	1	3.26 (1.93–4.59)
2007 (41)	All ages	Oxfordshire, UK	2004	395	2,701,909	10	…
2007 (42)	All ages	Takashima, Japan	1995	380	831,765	15	…
2007 (43)	All ages	Tartu, Estonia	2002	57	202,244	2	
2008 (44)	≥25	Mumbai, India	2006	68	313,722	2	…
2008 (45)	All ages	Melbourne, Australia	1998	151	613,262	2	1.23 (0.91–1.54)
2009 (46)	All ages	L'Aquila, Italy	1996	549	1,488,225	10	1.02 (0.84–1.20)
2009 (47)	All ages	South Indian	2005	40	462,938	0.5	…
2009 (48)	All ages	Joinville, Brazil	2005	94	974,094	2	2.35 (1.65–3.05)
2009 (49)	All ages	Mashhad, Iran	2006	79	450,229	1	0.85 (0.04–1.67)
2010 (50)	All ages	Ludwigshafen, Germany	2007	65	335,812	2	…
2010 (51)	All ages	Southern Italy	2001	24	77,470	2	2.37 (0–4.98)
2012 (52)	All ages	VaraŽdin, Croatia	2008	123	368,230	2	…
2012 (53)	All ages	Spain	2006	350	1,440,979	1	1.3 (1–1.58)
2012 (54)	All ages	Dublin, Ireland	2006	56	294,529	1	…
2013 (55)	All ages	Udine, Italy	2008	95	306,624		…
2013 (56)	All ages	Northern Portugal	1999	111	246,224	2	…
2013 (57)	All ages	Ludwigshafen, Germany	2008	152	838,285	5	…
2014 (58)	≥40	Hisayama, Japan	1995	53	32,854	12	…
2014 (59)	All ages	MediterraneanIsland, Greece	2011	25	86,436	1	…
2015 (60)	All ages	Southwestern Nigeria	2011	54	491,033	1	0.9 (0.1–1.72)
2015 (61)	≥16	Scotland, England	2011	128	695,335	1	…
2016 (62)	All ages	LAquila, Italy	2012	115	596,430	2	1.14 (0.6–1.69)
2017 (63)	≥35	Southern Region of Portugal	2015	82	280,081	1	1.69 (0.9–2.46)
2018 (64)	≥20	Northeastern Greece	2011	83	119,805	2	1.08 (0.2–1.94)

Among 52 studies, the incidence of ICH ranged from 8.6 per 100,000 to 161 per 100,000 person-years, among which the highest incidence of ICH was in China. A prospective study in Changsha, China (30) in 2004 showed that the incidence of ICH was 131 per 100,000 person-years. Followed by Greece, Japan, and four studies in Japan (25, 27, 42, 58), the incidence of ICH was all over 45/100,000, and the average incidence of ICH in Japan was 52.4/100,000 (95% CI: 35.7–69.2) person-years. In total, three studies regarding ICH were included in Greece: the incidence of ICH in southern Greece was as high as 46.5/100,000 person-years in 1998 (23), and the incidence of ICH in Greece was 29/100,000 person-years in 2014 (59), and as high as 69.3/100,000 person-years in 2018 (64); the average incidence of ICH in Greece was 50.3/100,000 person-years (95%CI: 7.9–92.7).

### Age subgroup analysis

Of the 52 studies included, 10 were age limits (22, 23, 25, 37, 39, 44, 59, 62, 64, 65). In total, two studies reported the incidence of ICH in patients aged ≥ 15 years (22, 39). One study aimed at patients aged ≥ 16 years (61). One article limited the study age to ≥18 (23). In total, two studies showed the incidence of ICH in patients aged ≥ 20 years (37, 64). Another study was aimed at patients aged > 25 years (44). The study population in another two studies was more than 35 years old (25, 63). Another study reported the incidence of ICH in people aged over 40 years (58). Excluding the aforementioned 10 studies, the incidence of ICH was 29/100,000 person-years (95% CI: 25.2–35.9), which was only 0.9/ 100,000 person-years, different from the total 52 studies without age restriction.

Among the 52 included studies (13, 14, 16–19, 21–27, 29, 32–36, 38–41, 44–49, 51, 56, 60, 62–64), 35 clearly showed the incidence of ICH in different age groups, among which three studies (17, 27, 64) were not included because age segments could not be combined with the other 32 studies. The incidence of ICH is concentrated in middle-aged and older adults, and it is rare in people under 45 years old (7) (Table 2). The incidence of ICH in people under 45 years old was 2.37/100,000 person-years (95% CI: 1.9–3), which is higher than that of 1.9/100,000 person-years (95% CI: 1.6–2.3) in a meta-analysis in 2010 (7).

**Table 2:** Incidence rate of ICH in different ages.

**Age**	**Numbers of patients**	**Person-years**	**Incidence/100,000**	**95% CI**	**Numbers of study**	**Incidence ratio (95% CI)**
<45 (13, 14, 17, 18, 20–25, 29, 32–36, 38, 40, 41, 44–49, 51, 57, 61, 63)	184	7,175,944	2.37	1.9–3.0	28	0.21 (0.1–0.32)
45–54 (13, 14, 18, 19, 21–26, 29, 32–36, 38, 40, 41, 44–49, 51, 57, 61, 63)	344	1,435,489	22.1	16.9–27.3	29	Reference
55–64 (13, 14, 17–19, 21–26, 29, 32–36, 38, 40, 41, 44–49, 51, 57, 61, 63)	640	1,164,530	55	43.8–66.2	30	2.63 (1.9–3.4)
65–74 (13, 14, 17–19, 21–26, 29, 32–36, 38–41, 44–49, 51, 57, 61, 63, 64)	1,159	1,043,790	111.3	90.7–132	32	5 (3.3–6.7)
75–84 (13, 14, 17–19, 21–26, 29, 32, 34–36, 38, 39, 41, 44–46, 48, 49, 51, 57, 61, 63, 64)	919	620,473	166	128.6–203.3	29	6.6 (4.8–8.4)
>84 (13, 14, 17–19, 21–26, 29, 32, 34–36, 38, 39, 41, 44–46, 48, 49, 51, 57, 61, 63)	427	763,816	95.4	79.5–111.3	28	9.7 (6–13.5)

ICH, intracerebral hemorrhage.

The investigation results showed that the incidence of hemorrhagic stroke increased exponentially with age, and the rate among men was higher than that among women (57, 60). A number of studies included in this review showed that the incidence of ICH increases with age and differs at the 85-year-old boundary. The incidence of ICH continued to increase with age in 17 studies (18, 21, 23, 25, 29, 32, 35, 38, 41, 44–46, 49, 51, 55, 60, 61). In seven studies (13, 14, 17, 22, 24, 36, 39), the incidence of ICH increased with age before 85 y but increased slowly or even decreased after 85 years. In total, five other studies (19, 20, 34, 48, 58) showed an increase in the incidence of ICH with age, except for the 75–84 years age group. As shown in Table 2, the integrated incidence of ICH increased with age and showed a downward trend after 85 years.

### Subgroup analysis of risk factors

In addition to the differences caused by the aforementioned factors, ICH incidence is affected by many risk factors. Therefore, the incidence of each risk factor for ICH is provided and summarized in Table 3. The incidence of ICH varies according to different risk factors; in a 2009 Brazilian study (8), the definition of hypertension was inconsistent with the international standard of blood pressure ≥ 140/90 mmHg; therefore, hypertension was not included as a risk factor in this study. In addition, many risk factors, such as overweight, peripheral artery disease, and physical activity intensity, were not included because of the small number of studies examining these.

**Table 3:** Ten common risk factors for ICH.

**Risk factors**	**Number of study**	**Number of patients**	**Person-years**	**Incidence/100,000 (95%CI)**	**Incidence ratio**
Hypertension (19, 26–28, 33, 36, 37, 39, 41, 52, 54, 56, 57, 61, 63)	15	2,064.9	9,784,791	28 (21.8–34.1)	Reference
Diabetes (19, 26–28, 33, 36, 37, 39, 41, 48, 52, 54, 56, 57, 61, 63)	16	376.9	10,295,947	4.1 (3.1–5.2)	0.2 (0.13–0.26)
Heart disease (19, 26, 28, 33, 36, 41, 48, 52, 54, 56, 57, 63)	12	497.4	7,691,988	7.1 (4.9–9.4)	0.27 (0.13–0.4)
Atrial fibrillation (19, 28, 36, 41, 48, 52, 54, 56, 57, 63)	10	83.4	6,000,825	1.6 (0.95–2.2)	0.1(0.04–0.16)
Smoking (19, 27, 28, 36, 37, 39, 41, 48, 52, 54, 57, 61, 63)	13	595.1	8,437,127	6.5 (4.7–8.4)	0.35 (0.23–0.47)
Excessive drinking (19, 27, 28, 36, 37, 61, 63)	7	200	2,954,459	6.2 (3–9.4)	0.32 (0.12–0.52)
Anticoagulant drugs (19, 26, 36, 48, 52, 54, 57, 61, 63)	9	112.6	4,528,027	2.3 (1.1–3.4)	0.17 (0.12–0.23)
Antiplatelet drugs (19, 36, 39, 48, 52, 54, 57, 61, 63)	9	273.7	5,214,520	5.5 (3.6–7.4)	0.33 (0.2–0.4)
Dyslipidemia (19, 28, 36, 39, 48, 52, 56, 57)	8	166.8	3,622,188	4.6 (2.6–6.6)	0.3 (.16–0.47)
Pre-TIA (19, 26, 36, 48, 52, 54, 56)	7	83	3,631,178	1.4 (0.4–2.3)	0.08 (0–0.23)

ICH, intracerebral hemorrhage.

**Figure 3:**
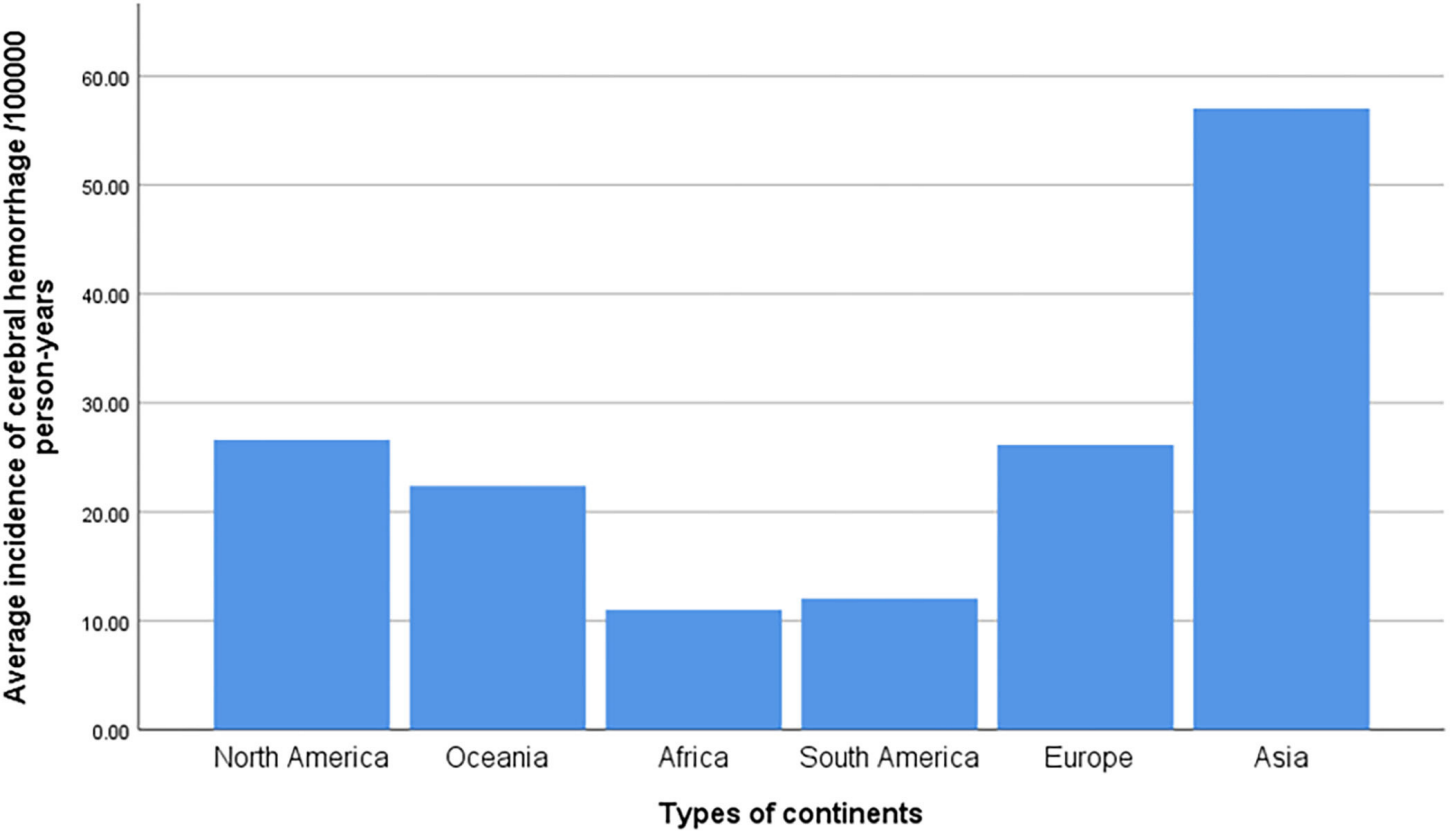
Random effect forest diagram of single incidence rate in ICH.

Of the 10 risk factors examined in this study, patients with hypertension had the highest incidence of ICH, followed by those with excessive alcohol consumption and heart disease. The incidence of ICH caused by transient ischemic attacks before ICH was the lowest. In 2004, the incidence of hypertensive ICH in Changsha was the highest, at 104.5/1,000, and in 2000, the incidence of hypertensive ICH in Japan also reached 41.8 per 100,000. Therefore, the incidence of ICH in the Asian population is much higher than in other continents, which may be closely related to the high incidence of hypertension (7, 66, 67). In a 2013 German study, the incidence of ICH caused by various risk factors (other than hypertension) was the highest in other countries, but the overall incidence of ICH in three German studies was low in most countries (Figure 3).

### Subgroup analysis at the site of bleeding

Under the influence of the vascular state, anatomical defects (ICH caused by factors such as arteriovenous malformation, intracranial aneurysm, and so on in patients, and the site of bleeding is easy to appear in the lenticular artery), and other multiple factors (such as cerebral vascular amyloidosis, severe fluctuations in blood pressure in patients with hypertension, and other vascular diseases under the action of different factors), the incidence of ICH was different for different bleeding sites. The incidence of lobar hemorrhage (16, 26, 37, 46, 58, 59, 62, 64) was 12.7/100,000 person-years (95%CI: 9.9–15.5). Because there have been the most studies on lobar hemorrhage, it was selected as the reference group at the bleeding site. As shown in Table 4, the basal ganglia region is the most common area for ICH and is associated with the highest incidence of ICH.

**Table 4:** Incidence rate of ICH in different locations.

	**Lobes (16, 26, 37, 46, 58, 59, 62, 64)**	**Brainstem (16, 26, 59)**	**Cerebellum (16, 26, 59)**	**Basal ganglia (16, 59)**	**Posterior fossa (46, 62)**	**Intracerebroventricular (16, 46, 57, 62)**
Number of patients	687	32	49	100	55	80
Person-year	5,412,745	1,683,399	1,683,399	543,399	2,084,655	2,762,857
Incidence	12.7 (9.9–15.5)	1.9 (0.3–3.5)	4.8 (1.4–8.3)	53.6 (0–135.9)	2.5 (1.4–3.5)	2.7 (0.5–4.8)
Number of study	9	3	3	2	2	4
Incidence ratio (95%CI)	Reference	0.39 (0–1.2)	0.28 (0.08–0.45)	2.7 (0–12)	0.23 (0–0.47)	0.62 (0–1.76)

ICH, intracerebral hemorrhage.

### Explanation of heterogeneity

The forest plot created in this study showed that the heterogeneity was as high as 97%, and the sensitivity analysis of the study conducted in Changsha, China, in 2004 was significantly biased. However, heterogeneity was only reduced by 1.8% when this study was excluded. Therefore, subgroup analysis and meta-regression were used to further analyze the sources of heterogeneity.

Subgroup analysis and meta-regression were performed on sex, age, risk factors, bleeding site, median year of the study, and study design. No significant sources of heterogeneity were found in the subgroup analysis. The publication year, median year of the study, and study design (prospective study and stroke registry) could explain 28.6% of the heterogeneity.

## Discussion

In summary, our main findings from the 52 studies concerning ICH, which were diagnosed by a neurologist and confirmed by autopsy, CT, or MRI, were as follows: First, the total incidence of ICH in 52 studies was 29.9/100,000 person-years (95% CI: 26.5–33.3), and the incidence did not decrease. The overall incidence of ICH remains high, and China had the highest incidence of ICH among the 52 included studies, followed by Greece and Japan. Two studies in China (33, 55) showed that the incidence of hypertension, the most important risk factor for ICH, was not very high in the Changsha area, and there was little correlation with the high incidence of ICH. The high incidence of ICH may be related to multiple factors, such as the gene (apolipoprotein) and the environment (68–70). In 2006, a study of ethnic differences in ICH showed that Asians and others (non-white New Zealanders) who immigrated to New Zealand had 1.5–3.0 times higher rates of ischemic stroke and primary ICH than New Zealand/European populations (39). The high incidence of ICH in China warrants further investigation. Greece has the highest incidence of ICH in Europe, which may be related to the older population included in the Greek study and the high incidence of hypertension, diabetes, coronary heart disease, and other diseases (24, 63). The high consumption of antioxidants (Mediterranean diet) in the population leads to a low incidence of atherosclerosis-related cerebral infarction, and a high incidence of hypertensive ICH may be among the reasons for the high incidence of ICH in Greece (24, 71–73). The high incidence of ICH in Japan may be related to the aging population and an increasingly westernized lifestyle (56, 74). Season is an important factor in the occurrence of ICH (27).

Second, the incidence of ICH in men is higher than in women, as shown in most studies included in this review. The incidence of ICH in men is significantly higher than that in women in the studies of Greece (23, 64), Japan (27), Changsha, China (33), Chile (36), Italy (51), Croatia (52), Spain (53), and Portugal (63). However, in studies conducted in Germany (24) and Sweden (26), the incidence of ICH in women was higher than in men. The difference in the incidence of ICH caused by sex differences is closely related to age (75), but it is generally affected by social pressure, dietary habits, genes, and multiple other factors (76–80).

Third, hypertension is the most common risk factor for ICH, and the incidence of ICH in the hypertensive population is 28/100,000 people per year, which is very close to the total ICH incidence of 29.9/100,000 people per year. However, as the use of antihypertensive drugs and people's awareness of hypertension (57) improved (81), the incidence and prevalence of hypertension also showed different degrees of change. Factors such as diabetes, antiplatelet and anticoagulant drugs, dyslipidemia, smoking, and excessive alcohol consumption also play an important role in the incidence of ICH.

The incidence of ICH increases with age but decreases with age > 85 years. Aging is an irreversible process and a risk factor for ICH. With increasing age, the incidence of hypertension, atherosclerosis, and aneurysms increases, which may contribute to the onset of ICH. These rising risk factors are important reasons for the rapid increase in ICH incidence in middle-aged and older adults. However, there is a decrease in the incidence of ICH in people older than 85 years, which may be partly explained by death from other conditions. Moreover, older adults often do not want to visit hospitals for CT examinations, and some refuse autopsies (13).

Fifth, the incidence of ICH showed an increasing trend among younger patients, and the proportion of young people among the total number of patients with ICH increased. The trend of younger patients with ICH has been reported by many researchers (7, 82). Although the mortality rate associated with ICH in young people is not high, it is more likely to cause long-term disability due to hemorrhage (10, 82). In the past, we first focused on middle-aged and older adults with a high incidence of cerebrovascular diseases, so the formulated treatment and prevention programs are aimed at middle-aged and older adults. There is a lack of targeted and normative treatments for ICH in young people, which requires further research.

A large amount of research data were included in this study to summarize the epidemiological changes in ICH over the past 40 years. The data were extensive, highly representative, and widely applicable. It is of great practical value to study the changing trend of sex and age in the incidence of ICH, which is of great significance to the prevention and treatment of ICH.

However, several limitations remain. First, of the 52 studies included in this study, 32 were conducted in Europe, and the number of studies conducted on other continents was small, especially in Africa, from which only one article was included; therefore, the included studies may not be representative of the global situation. However, the incidence of ICH is highest in Asia, and China and Japan have the highest incidence in Asia, consistent with previous studies (83, 84). Moreover, regional differences in the incidence of ICH in Africa need to be studied further. Second, due to the varying level of information on ICH provided by various studies, the age, sex, economic status, and other factors of ICH in each study cannot be accurately obtained, and the incidence of ICH not be adjusted. Therefore, we used the crude ICH incidence in this study. The heterogeneity of the forest map in the meta-analysis was as high as 97%. As this study was a single-rate meta-analysis and lacked a control group, the heterogeneity was much higher than that of the intervention and diagnostic experiments. Therefore, the data were adjusted by double inverse sine conversion, and sensitivity, subgroup, and meta-regression analyses were performed on the sources of heterogeneity. However, no obvious source of heterogeneity was identified. Finally, the incidence of ICH before 45 years and after 85 years is old at least partly related to the small sample size. However, we calculated the relative incidence rate of each age group using the rate of the 45–55-year-old population as a reference, so the small sample size had little effect on the results.

Research on the incidence of ICH in the past 40 years, from 1980 to 2020, shows that the incidence of ICH has not decreased worldwide, and the prevention and treatment of ICH still need to be improved continuously according to age, sex, risk factors, and other factors. Targeted and normative strategies should be gradually developed in the future.

## Data availability statement

The original contributions presented in the study are included in the article/supplementary material, further inquiries can be directed to the corresponding author/s.

## Author contributions

LZ, SW, and X-LZ designed the research and determined the structure of the manuscript. SW, X-LZ, TY, JM and L-XX selected the references and contributed to the writing. SW, X-LZ, TY, and Y-PZ collected the data. SW, L-XW, and H-FZ helped to analyze the results of this meta-analysis. LZ, X-LZ, SW, and YZ contributed to the revision and finalization of the article. All the authors contributed to the article and approved the submitted version.

## Funding

This study was supported by the National Science and Technology Fundamental Resources Investigation Program of China to LZ (No.2018FY100900), the Hunan Provincial Natural Science Foundation of China Grant to YiZ (No.2021JJ30923), the Provincial Science and Technology Innovation Leading Talents Project to LZ (No.2021RC4014), and the National Clinical Research Center for Geriatric Disorders (Xiangya Hospital).

## Conflict of interest

The authors declare that the research was conducted in the absence of any commercial or financial relationships that could be construed as a potential conflict of interest.

## Publisher's note

All claims expressed in this article are solely those of the authors and do not necessarily represent those of their affiliated organizations, or those of the publisher, the editors and the reviewers. Any product that may be evaluated in this article, or claim that may be made by its manufacturer, is not guaranteed or endorsed by the publisher.
